# Parapneumonic empyema diagnosed by chest radiograph and computed tomography

**DOI:** 10.3402/jchimp.v3i1.20503

**Published:** 2013-04-17

**Authors:** Ning Jin, John Paul Brady, David M. Widlus

**Affiliations:** 1 Union Memorial Hospital, Baltimore, MD, USA; 2 American University of the Caribbean School of Medicine, Coral Gables, FL; 3 Department of Radiology, University of Maryland School of Medicine, Baltimore, MD, USA

**Keywords:** chest x-ray, pneumonia, empyema, chest tube, chest CT scan

## Abstract

Pleural effusion is commonly seen associated with pneumonia. When this progresses to empyema, directed therapy is frequently required. Chest radiographic and computed tomography findings can help distinguish empyema from a transudative pleural effusion.

A 55-year-old African American male with a history of HIV on antiretroviral therapy, hepatitis C, hypertension, and uncertain tuberculosis status presented with pleuritic chest pain, gradually worsening over 2 weeks, accompanied by a productive cough with cupful volumes of yellowish, foul-smelling sputum. He noted dyspnea on exertion and chills, but denied fever or headache. The patient denied alcohol usage but had a multi-year half pack per day history of cigarette smoking. Heroin and cocaine use 10 years earlier was noted. The patient was afebrile with an oxygen saturation of 96% on room air. Pertinent physical findings included decreased bilateral breath sounds, bibasilar crackles, dullness to percussion in the left lower lobe, and +1 edema in the lower extremities. The patient's home medications included abacavir–lamivudine 600–300 mg daily, raltegravir 400 mg BID, tenofovir 300 mg daily, and methadone 40 mg daily.

Significant laboratory findings included, WBC 10,200, neutrophil 79.8%, Plt 39,000, PT 19 sec, and INR 1.6. The patient's initial chest x-ray showed a left pleural effusion with an air-fluid level (Fig. 1). A computed tomography (CT) scan demonstrated a loculated fluid collection in the left pleural space with a large volume of gas felt to indicate an empyema (Fig. 2). Sputum Gram stain showed rare Gram-positive cocci in chains, negative for acid fast bacilli. Peripheral blood culture was negative.

**Fig. 1:**
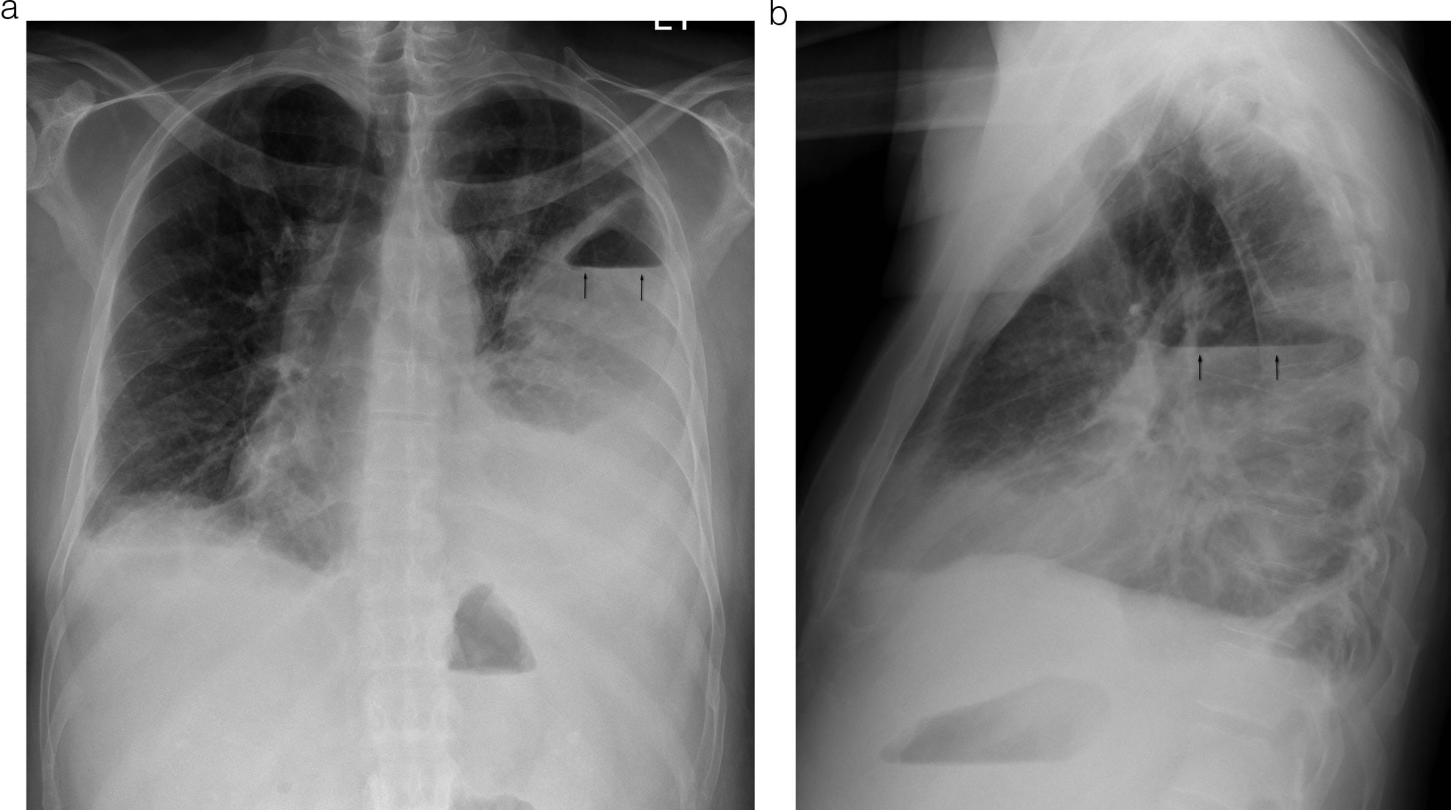
(a) PA and (b) lateral views of the chest demonstrate a large left pleural collection with an air-fluid level (arrows). Small right pleural effusion is also seen.

**Fig. 2:**
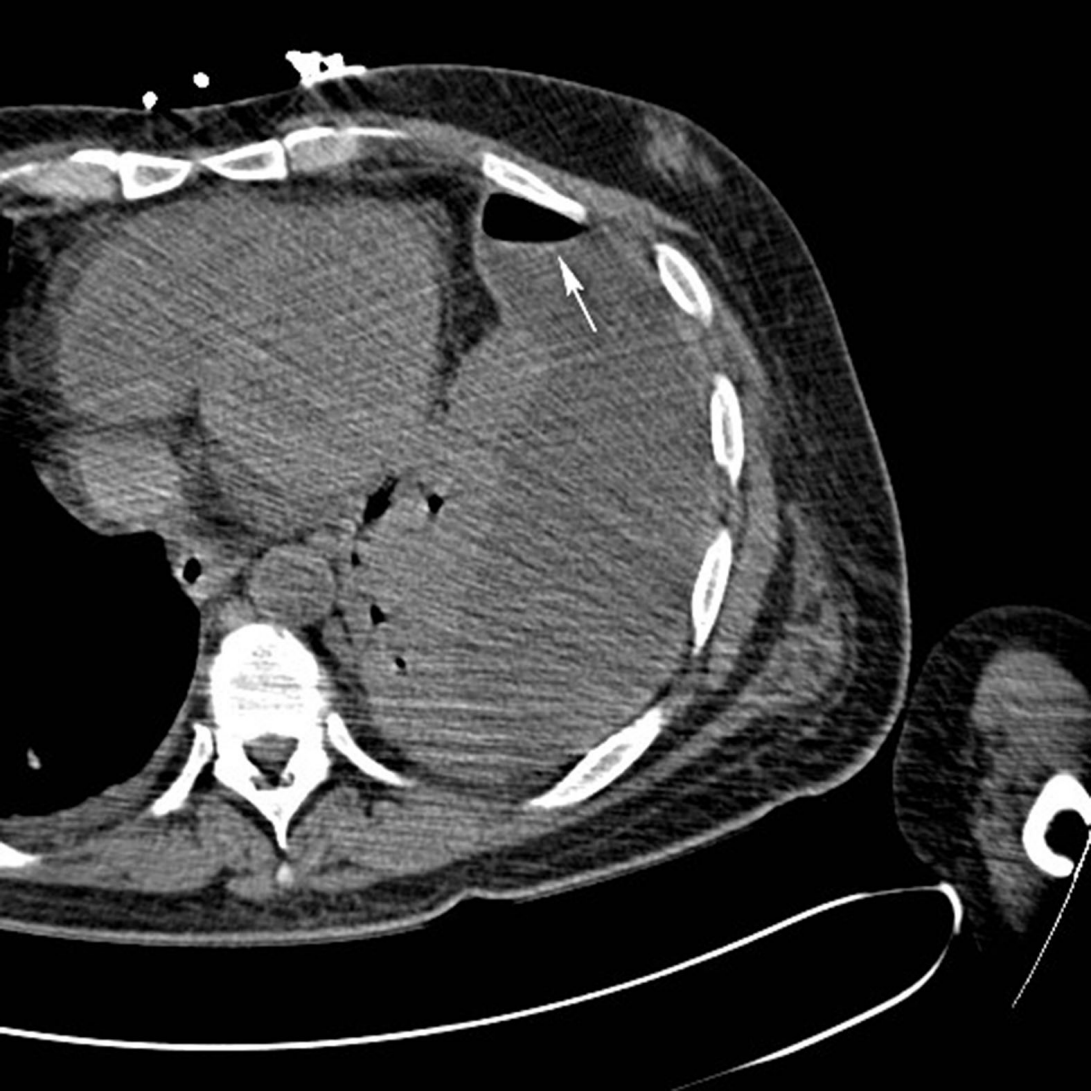
CT scan of the chest showing the loculated left pleural fluid collection with air-fluid level (arrow) and the adjacent parenchymal consolidation.

The patient was started on vancomycin 1 g IV q12 h and piperacillin–tazobactam 3.375 mg IV q6 h. He underwent CT-guided chest tube placement by the interventional radiology service (Fig. 3). The pleural fluid culture revealed peptostreptococcus and *Campylobacter gracilis*. The chest tube drained a total of 700 ml of purulent fluid. Chest tube removal was possible after 1 week. Vancomycin and piperacillin–tazobactam were discontinued and a 2-week course of oral clindamycin began. The patient's subsequent chest CT scan showed resolution of the left empyema with marked improvement in consolidation in the left lower lung (Fig. 4). The patient was discharged on his home medications and clindamycin with a follow-up appointment at the infectious diseases clinic.

**Fig. 3:**
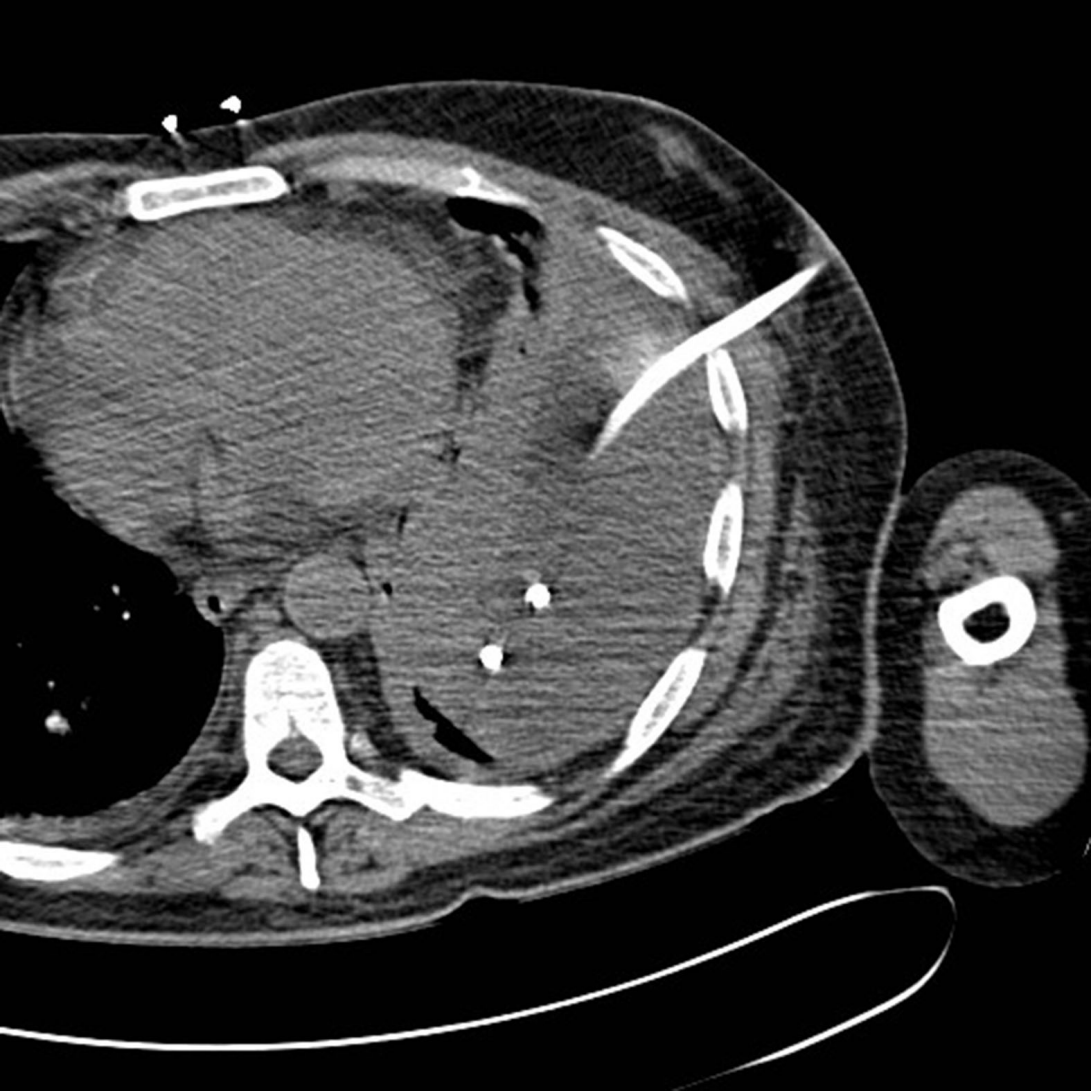
Small bore chest tube was placed from an anterior approach.

**Fig. 4:**
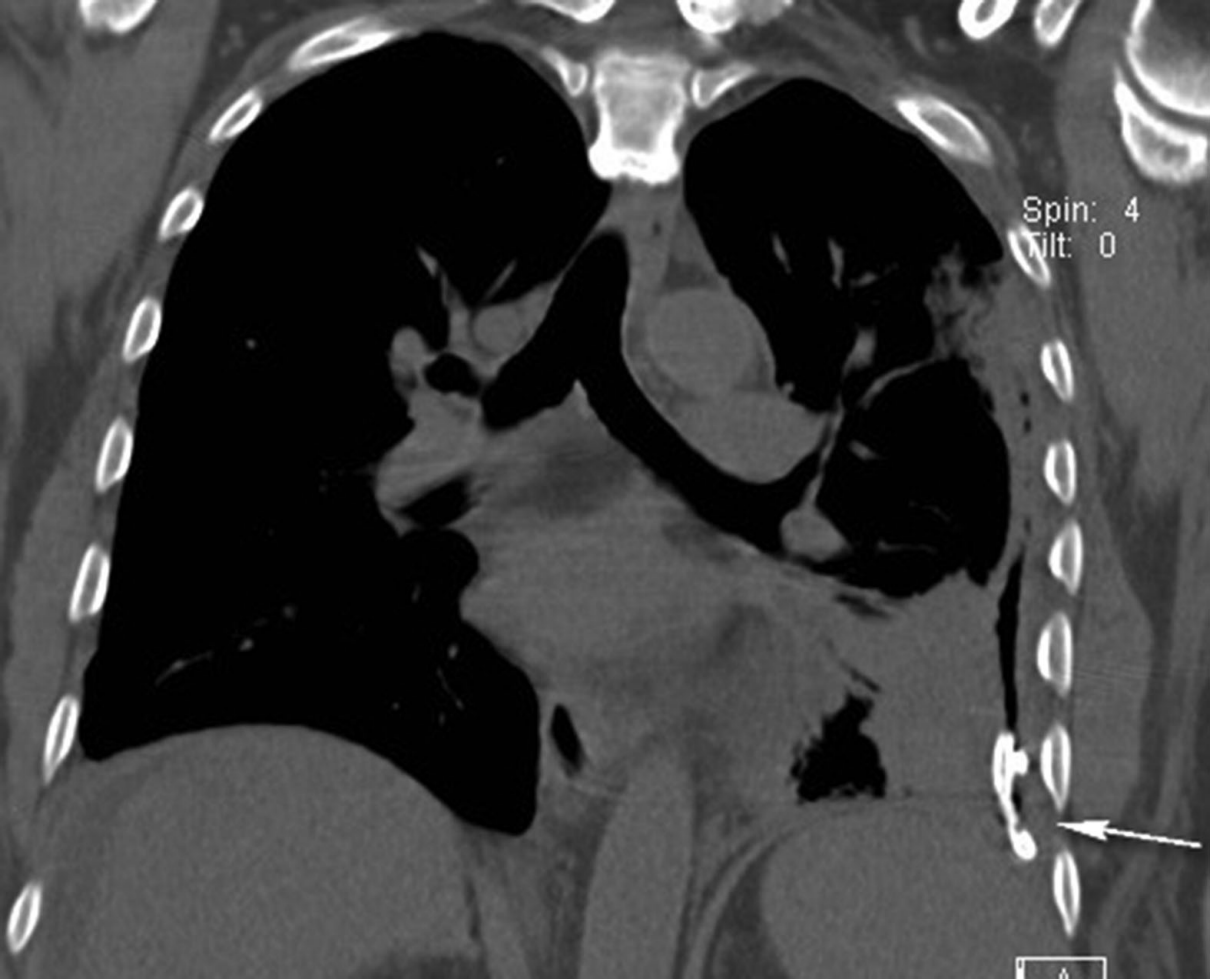
Coronal image of the chest demonstrates a small bore chest tube partially surrounded by air in the left pleural space (arrow). No remaining fluid is seen. Adjacent lung consolidation has decreased.

Over one million patients in the United States are hospitalized annually with pneumonia and in one-third to two-third of patients, pleural effusions occur. The majority of these are resolved by treating the underlying pneumonia. However, 10–20% progress to an exudative stage. Subsequent fibrinopurulent and organizing phases can then be seen with the requirement for more aggressive treatment. Imaging characteristics of empyema typically include a loculated appearing fluid collection with a thick, enhancing rim and increased central radiodensity compared to simple fluid (higher protein content in exudates versus transudates). A large air-fluid level, as was seen in this case, is less common and more likely to be seen with a bronchopleural fistula. Treatment options include chest tube drainage, which is most successful for thin exudative collections, fibrinolysis, which is most successful for collections found in the fibrinopurulent phase before the organizing phase, and decortication, either open or using video-assisted thoracoscopy. Mortality, which occurs in up to 33% based on age and co-morbidity, is substantially reduced by early treatment.
